# Draft Genome Sequences of Six Actinobacillus pleuropneumoniae Serotype 8 Brazilian Clinical Isolates: Insight into New Applications

**DOI:** 10.1128/genomeA.01585-14

**Published:** 2015-03-05

**Authors:** Monalessa Fábia Pereira, Ciro César Rossi, Fabíola Marques de Carvalho, Luiz Gonzaga Paula de Almeida, Rangel Celso Souza, Ana Tereza Ribeiro de Vasconcelos, Denise Mara Soares Bazzolli

**Affiliations:** a Departamento de Microbiologia, Laboratório de Genética Molecular de Micro-organismos, Instituto de Biotecnologia Aplicada à Agropecuária–BIOAGRO, Universidade Federal de Viçosa- UFV, Viçosa, Minas Gerais, Brazil; b Laboratório de Bioinformática, Laboratório Nacional de Computação Científica, LNCC, Petrópolis, Rio de Janeiro, Brazil

## Abstract

Actinobacillus pleuropneumoniae is the causative agent of swine pleuropneumonia, a highly contagious disease associated with pigs of all ages that results in severe economic losses to the industry. Here, we report for the first time six genome sequences of A. pleuropneumoniae clinical isolates of serotype 8, found worldwide.

## GENOME ANNOUNCEMENT

Actinobacillus pleuropneumoniae is the causative agent of porcine pleuropneumonia, a costly, severe, and highly contagious infectious disease (1). There are 15 known serotypes of this microorganism, the prevalence of which varies greatly worldwide (2). Serotype 8 isolates, the most widespread in southeastern Brazil (3), display high cytotoxic activity and are generally responsible for low mortality but high morbidity. This serotype is also dominant in other pig-producing countries, such as Mexico and the United Kingdom (4, 5).

We sequenced six serotype 8 A. pleuropneumoniae clinical isolates that are persistent in farms in southeastern Brazil and present different virulence degrees (6). Each isolate was grown in culture medium (BHI, BD Biosciences), and total genomic DNA was extracted using the FastDNA Spin kit (MP Biomedicals). Whole-genome sequencing was performed using an Ion Torrent personal genome machine sequencer (Life Technologies) using 200-bp chemistry with an Ion 318C chip. Assembly for each isolate was performed with Newbler version 2.9. Contigs were reordered with Mauve using A. pleuropneumoniae JL03 as the reference genome (GenBank accession no. CP000687). Automatic annotation of genes predicted with the GeneMark and Glimmer programs was performed by BLASTp against the NCBI NR, KEGG, UniProt, and TCDB databases using 100% query and subject coverage and a minimum of 95% positive as the cutoff. Manual annotation was performed using the System for Automated Bacterial Integrated Annotation (SABIA) (7).

Genome sequencing of A. pleuropneumoniae isolates 460, 518, 597, 780, 1,022, and 5,651 resulted in high-coverage assemblies of the 2.24 ± 0.03 Mbp genomes (between 90- and 123-fold), with 40.33% ± 0.31% GC content and an average coding gene length of 844.97 ± 9.26 bp. The coding regions correspond to 82.41% ± 0.58% of the genome, which should represent most of the functionally annotated genes and allow for comparative studies using these sequences. Approximately 2,358.16 ± 42.01 loci were predicted for each genome, and these showed significant hits with databases of functional data: KEGG (90.50%), UniProt (79.40%), NCBI NR (93.03%), and TCDB (23.55%). Gene prediction revealed 48.17 ± 1.94 tRNA genes and four rRNA operons. According to a Bidirectional Best Hit comparison, the genomes present 2,296 ± 33.32 clusters in common with strains JL03 and L20 (GenBank accession no. CP000569) and 79.5 ± 24.05 nonclustered proteins, revealing high genetic variability of this species, often associated with intrinsic and environmental factors, such as increased horizontal gene transfer frequency, because of the remarkable ability of A. pleuropneumoniae to transform naturally (8, 9).

These new six A. pleuropneumoniae genome assemblies are the first for serotype 8 to be published in the major sequence databases. Because of the relevance of this serotype in many countries, the public availability of these sequences will be globally helpful for comparative genomic studies, thereby contributing to the discovery and identification of common virulence determinants and molecular markers that could be used for effective diagnosis and vaccine production.

### Nucleotide sequence accession numbers.

The assembled genomes of the six A. pleuropneumoniae isolates have been deposited at the NCBI database under the accession numbers JSVF00000000, JSVG00000000, JSVV00000000, JSVX00000000, JSVY00000000, and JSVZ00000000.
